# One stone for two birds: Endophytic fungi promote maize seedlings growth and negatively impact the life history parameters of the fall armyworm, *Spodoptera frugiperda*

**DOI:** 10.3389/fphys.2023.1253305

**Published:** 2023-10-12

**Authors:** Sharon W. Kinyungu, Ayaovi Agbessenou, Sevgan Subramanian, Fathiya M. Khamis, Komivi S. Akutse

**Affiliations:** ^1^ International Centre of Insect Physiology and Ecology (icipe), Nairobi, Kenya; ^2^ Julius Kühn Institute (JKI) - Federal Research Centre for Cultivated Plants, Institute for Biological Control, Dossenheim, Germany; ^3^ Center for Development Research (ZEF), Department of Ecology and Natural Resources Management, University of Bonn, Bonn, Germany; ^4^ Unit of Environmental Sciences and Management, North-West University, Potchefstroom, South Africa

**Keywords:** fungal endophytes, plant growth, life-history parameters, fall armyworm, biological control

## Abstract

The fall armyworm (FAW) *Spodoptera frugiperda*, is a voracious pest of cereals native to the Americas and which invaded Africa in 2016. Chemical control is the main management option, which however remains ineffective and unsustainable. Fungal endophytes are increasingly used as alternative for the management of insect pests of economic importance. This study assessed the potential of eight endophytic fungal isolates to colonize maize plant and their ability to promote seedlings growth through seed and foliar inoculations, as well as their suppressive effects on FAW. Fungal colonization rates of different plant parts by the endophytes varied as per the inoculation methods. *Beauveria bassiana* ICIPE 279 colonized more than 60% of all the seedling parts while *B*. *bassiana* G1LU3 only colonized stem (25%) and leaf (5%) tissues through foliar inoculation. *Trichoderma atroviride* F2S21, *T. asperellum* M2RT4, *T. harzianum* F2R41, *Trichoderma* sp. F2L41, *Hypocrea lixii* F3ST1 and *Fusarium proliferatum* F2S51 successfully colonized all the plant parts and therefore were selected and further evaluated through seed inoculation for their endophytic persistence, effect on plant growth, and pathogenicity to *Spodoptera frugiperda* immature and adult stages. Weekly assessment showed varied effect of the endophytes on maize plant growth parameters compared to the control. During the first week, percentage colonization of the plant parts ranges between 90%–100%, 65%–100%, and 60%–100%, in the roots, stems, and leaves, respectively for all the five tested isolates. However, the colonization pattern/rates significantly decreased over time for *H. lixii* F3ST1 in the stems and leaves, and for *T. harzianum* F2R41 in the leaves and for *T. asperellum* M2RT4 in the roots. In addition, *T. harzianum* F2R41 outperformed all the other isolates in boosting the plant height, whereas *H. lixii* F3ST1 and *T. asperellum* M2RT4 outperformed all the other isolates in increasing the wet and dry shoots weight. Furthermore, the number of egg masses laid on endophytically-colonized maize plants varied among the treatments. *Trichoderma asperellum* M2RT4 and *H. lixii* F3ST1 endophytically-colonized maize plants significantly reduced the number of egg masses and the defoliation/feeding rates of the pest compared to the control. Additionally, *T. harzianum* F2R41 had the highest negative impact on the pupation and adult emergence of *S. frugiperda* with a female-biased sex ratio. Our findings indicate that *T. asperellum* M2RT4, *T. harzianum* F2R41, and *H. lixii* F3ST1 hold a potential to be developed as endophytic-fungal-based biopesticides for sustainable management of *S. frugiperda* and as plant growth promoters.

## 1 Introduction

The invasive fall armyworm (FAW), *Spodoptera frugiperda* (J. E. Smith) (Lepidoptera: Noctuidae), is a destructive insect pest originated from the Americas that was first reported outside its region of origin in 2016 in West Africa (Goergen et al., 2016). Subsequently, the pest has quickly spread across Africa (Koffi et al., 2021; Niassy et al., 2021) attacking maize, the continent’s most staple food crop with damage estimated at US$2 billion per year (Timilsena et al., 2022). Additionally, the pest displays an enormous destructive potential attacking other host plants including sorghum, rice, sugarcane as well as a variety of vegetable crops and as a result causing tremendous yield losses and therefore posing significant threat to food security and the livelihoods of millions of smallholder farmers in the newly invaded countries (Babendreier et al., 2020; Koffi et al., 2021; Guimapi et al., 2022). Efforts to control FAW in sub-Saharan Africa largely depend on frequent application of chemical insecticides which has been ineffective due to the pest’s concealed feeding behavior (Harrison et al., 2019). Besides, the overreliance on chemical insecticides has led to the development of resistance among FAW populations (Nagoshi et al., 2021; Sokame et al., 2022). Also, there is a continuous growing concern regarding environmental and human health risks towards these chemical insecticides which make this strategy less attractive (Agboyi et al., 2023). As a result, environmentally friendly control strategies are being promoted as alternatives for sustainable management of this voracious and polyphagous pest (Paola et al., 2018). So far, other non-chemical FAW control methods such as biological control have mainly focused on the use of parasitoids and entomopathogenic fungi (Akutse et al., 2019; Sokame et al., 2020; Mohamed et al., 2021).

Advantages of using entomopathogenic fungi reside in the fact that they are environmentally friendly, very safe for humans and are not harmful to other groups of beneficial organisms such as arthropod natural enemies (Akutse et al., 2020). However, considering the environmental effects on the inundative application of these entomopathogenic fungi and the versatility of microbial agents (Vega, 2018), their use as endophytes for sustainable management of insect pests and plant pathogens has also become a new prominent area of interest under the continuous climate change scenario (Barelli et al., 2016). Endophytic fungi are an example of microorganisms whose presence within host plants through symbiotic interaction provides multiple benefits (Jaber, 2018). Firstly, using different method of inoculation (seed treatment, soil drench or foliar spray) (Tefera and Vidal, 2009; Bamisile et al., 2018), the presence of endophytic fungi in the host plant tissues following successful colonization triggers a systemic resistance mechanism against insect pests and plant diseases (Paradza et al., 2021; Agbessenou et al., 2022). Secondly, endophytic fungi are a potential reservoir of secondary metabolites (Evidente et al., 2009) assisting their host plants in fighting against herbivorous insect pests, their vectored pathogens and diseases (Barelli et al., 2016; Poveda et al., 2020). Thirdly, they participate/facilitate in nutrient uptake from the soil to the host plants, contributing to promote plant growth (White et al., 2019). Besides, the use of endophytic fungi for the management of insect pests and diseases of economic importance, and as plant growth promoters, they are also another important component of integrated pest management (IPM) programs which is generally assumed to be compatible with other pest control approaches (Akutse et al., 2020).

Since the invasion of FAW in sub-Saharan Africa, attention was mainly focused on applying potent entomopathogenic fungal isolates through inundative approaches against both adult and immature stages of the pest (Akutse et al., 2019; Akutse et al., 2020). However, there is a limited information regarding the emerging multiple roles played by fungal entomopathogens and some endophytic fungal isolates such as *Trichoderma* species in sustainably managing the fall armyworm while also promoting growth of maize plant. Therefore, to overcome the environmental effects in inundative application of these entomopathogens and reduce the cost of application, the objectives of this study were, to investigate the ability of some fungal isolates to endophytically colonize maize host plant, then evaluate the potential of the best performing isolates as plant growth promoters and determine their effect on the life history parameters of *S. frugiperda*.

## 2 Materials and methods

### 2.1 Fungal cultures

Eight fungal isolates [4 *Trichoderma*–*Trichoderma atroviride* F2S21 and *T. harzianum* F2R41 (isolated from onion), *Trichoderma* sp. F2L41 and *T. asperellum* M2RT4 (from monocots), 2 *Beauveria*–*B. bassiana* G1LU3 (from monocots), *B. bassiana* ICIPE 279 (from coleopteran larva), *Fusarium proliferatum* F2S51 (from onion), and *Hypocrea lixii* F3ST1 (from maize)] were used in this study. All the isolates were obtained from the International Centre of Insect Physiology and Ecology (*icipe*)’s repository of pathogens maintained at Arthropod Pathology Unit Germplasm and screened for their potential to colonize maize plants through seed inoculation and foliar application with conidia suspension. They were cultured on potato dextrose agar (PDA) (OXOID CM0139, Oxoid Ltd., Basingstoke, United Kingdom) in 10 cm diameter Petri dish plates and incubated at 25°C ± 2°C (Muvea et al., 2014). Fungal suspensions of each isolate were prepared by harvesting conidia with a sterile spatula from two-to three-week-old sporulated plates and suspending them in sterile distilled water containing 0.05% Triton X-100 in 10 mL universal glass bottles with three glass beads (3 mm) (Fargues et al., 1992). The resulting suspension was vortexed for 5 min to obtain a homogenous conidial suspension (Akutse et al., 2019). Conidial concentration of each fungal strain was determined using a Neubauer hemocytometer (VWR International, United States) and adjusted to 1 × 10^8^ conidia/mL through serial dilutions prior to inoculation of seeds (Inglis et al., 2012).

To assess the conidial viability of the fungal strains, 100 µL of the 3 × 10^6^ conidia/mL suspension was inoculated onto four fresh PDA plates for each isolate prior to each experiment (Inglis et al., 2012). Inoculated plates were sealed with Parafilm membrane and incubated in complete darkness at 25°C ± 2°C. At 18 h post-incubation, four sterile microscope coverslips were placed randomly on the surface of each inoculated plate (Inglis et al., 2012). The percentage germination was determined by counting 100 randomly selected conidia under each coverslip under a light microscope at ×400 magnification (Leica DM500) (Akutse et al., 2019). Conidia were considered as germinated if the germ tube was at least twice the size of the spore (Inglis et al., 2012). Each plate served as a replicate with four replicates per isolate.

### 2.2 Maize inoculation with entomopathogenic fungi

Maize seeds (*Zea mays* L.) variety SC 403 obtained from Seed Co. Kenya, Nairobi, Kenya, were used for all experiments.

#### 2.2.1 Maize seed inoculation

Maize seeds were surface sterilized in 70% ethanol for 2 min and then immersed for 3 min in 1.5% sodium hypochlorite (NaOCl). After three rinses in sterile distilled water, the surface sterilized seeds were placed on sterile filter paper in a laminar flow cabinet until the residual water evaporated (Akutse et al., 2013). To confirm the effectiveness of the surface sterilization process, the third rinse water was spread on PDA media and plate imprinting was also conducted (Inglis et al., 2012). The sterilized seeds were then soaked in the fungal suspension containing 1 × 10^8^ conidia/mL of each fungal strain or in 0.05% Triton X–100 solution for 2 h. Inoculated and control seeds were then transferred into plastic pots (8 cm diameter × 7.5 cm high) containing autoclaved planting substrate. The substrate was a mixture of manure and soil at the ratio of 1:5, which was sterilized in an autoclave for 2 h at 121°C and was allowed to cool for 72 h prior to sowing (Akutse et al., 2013; Jaber and Enkerli, 2016). For each treatment, four seeds were inoculated and sowed per pot and pots were maintained in greenhouse conditions for 14 days. Subsequently, after germination, one plant (inoculated or uninoculated) per plastic pot was used for each treatment/assessment. Treatments (control and inoculated plants) were arranged in a completely randomized design (CRD) and replicated four times over time.

#### 2.2.2 Foliar inoculation

For foliar inoculation, fungal conidia were harvested under sterile conditions and adjusted to a concentration of 1 × 10^8^ conidia/mL as described above. Two-week old seedlings raised from uninoculated maize seeds were inoculated by spraying the adaxial surface of all the fully expanded leaves of each plant with 10 mL of conidial suspension using a hand-held plastic sprayer (Russo et al., 2015). The control plants were sprayed with sterile distilled water containing 0.05% Triton X-100. One plant (inoculated or uninoculated) per plastic pot was used for each treatment/assessment. Treatments (uninoculated and inoculated plants) were arranged in a completely randomized design (CRD) and replicated four times over time.

### 2.3 Colonization assessment

Two weeks following seed and foliar inoculation of maize seedlings with fungal isolates, systemic plant colonization by the inoculated fungal strains was determined through destructive sampling (Russo et al., 2021). The seedlings (after thorough washing of the roots with tap water) were divided into three different sections (1 cm^2^): roots, stems, and leaves using a sterile scalpel. Individual parts were surface-sterilized by submerging for 2 min in 70% ethanol, followed by 2 min in 1.5% sodium hypochlorite and rinsed thrice in sterile distilled water (Akutse et al., 2013; Russo et al., 2021). Plant materials were then dried on sterile filter paper in laminar flow cabinet. Five pieces were sampled from each plant tissue and then evenly plated onto PDA media amended with a 0.05% antibiotic (streptomycin sulphate salt) (Russo et al., 2021). In addition, the last rinse water was plated on PDA and plate imprinting was also conducted to assess the effectiveness of the surface sterilization process of the plant materials (Akutse et al., 2013; Sun et al., 2018). All Petri dishes were incubated at 25°C ± 2°C in complete darkness and inspected every 2, 3 days for 10 days to observe and record fungal outgrowth (Akutse et al., 2013). The identification of fungal outgrowth from the plated plant samples was based on the morphological characteristics and microscopic examination of conidia through slides preparation in comparison with the mother plates according to Koch’s postulates (Petrini and Fisher, 1986; Agbessenou et al., 2020). Only positive colonization by the inoculated endophytes were recorded (Muvea et al., 2014). Treatments were arranged in a completely randomized design (CRD) replicated four times. Colonization rate (%) of the plant parts was calculated as follows:

$$Colonization\;\left(\%\right)=\frac{Number\;of\;plant\;tissue\;pieces\;exhibiting\;fungal\;outgrowth}{Total\;number\;of\;plant\;tissue\;pieces\;plated\;out}\times 100$$

### 2.4 Endophyte persistence and assessment of plant growth parameters in maize

The outcome of the inoculation method showed that seed inoculation technique was the most effective for colonization of maize plant tissues and was therefore used for subsequent bioassays (unpublished data). Five of the isolates (*T. atroviride* F2S21, *T. harzianum* F2R41, *Trichoderma* sp. F2L41, *T. asperellum* M2RT4, and *H. lixii* F3ST1) that successfully colonized maize plant part tissues through seed inoculation were selected and used for the subsequent experiments. To examine the fungal colonization persistence and the effect of these isolates on the plant growth promotion parameters, seed inoculation was conducted with the above selected endophytes and control (endophyte-free) as described in the colonization experiment, where the plants were grown in plastic pots (8 cm diameter × 7.5 cm high) and which were arranged in a completely randomized design (CRD). Colonization was assessed weekly for 5 weeks starting at 1-week post-germination through destructive sampling where each weekly sampling consisted of new plants from four different pots (each pot used as replicate) (Paradza et al., 2021). After recording the growth parameters, the plants were uprooted for the colonization assessment. The following recorded growth parameters were plant height (the distance from plant base to the tip-which is the uppermost leaf with a visible leaf collar), number of unfolded leaves, leaf length (bottom to its tip), leaf width (at the widest part of the leaf) and plant dry weight/biomass (after drying for 24 h at 60°C) (14, 20). The fresh and dry shoot weights were also measured only in the final week to assess the total accumulated shoot biomass for the entire growing period (Paradza et al., 2021).

### 2.5 Insect rearing

*Spodoptera frugiperda* rearing was carried out according to the methodology developed by Akutse et al. (2019). The colony was established with specimens collected on maize crops from Siaya and Homa Bay counties (−0.61401° Latitude, 34.09095° Longitude; 1,215 m a.s.l.), Kenya in April-July 2017 (Akutse et al., 2019). The field-collected larvae were reared on semi-synthetic diet (Prasanna et al., 2018; Akutse et al., 2019) until pupation. The pupae were kept in ventilated Perspex cages (40 × 40 × 45 cm) until adult emergence; and upon emergence, moths were fed with 10% sugar solution soaked in balls of cotton wool. Four potted maize plants were introduced in the cages for oviposition. Subsequently, the plants were removed after 24 h and transferred to separate ventilated cage (50 × 50 × 60 cm) for egg hatching (Akutse et al., 2019). Leaves with neonate larvae were removed from these plants, 3 days after the larvae hatched and kept into clean cages (50 × 50 × 60 cm) lined with paper towel to absorb excess moisture and fine netting infused lid for ventilation. The larvae were supplied daily with fresh maize leaves as food until they pupated (Akutse et al., 2019). Larvae were reared separately (or in very few numbers for large cages) from the third instar onwards to prevent cannibalism. The pupae were collected from the cages using a fine camel hair brush, sexed and 100 pupae were placed at a ratio of 1:1 (male: female) inside individual sleeved Perspex cages (40 × 40 × 45 cm) for adult emergence.

### 2.6 Effect of endophytic colonization of maize on life history parameters of *Spodoptera frugiperda*

Five of the endophytic isolates (*T. atroviride* F5S21, *T. harzianum* F2R41, *Trichoderma* sp. F2L41, *T. asperellum* M2RT4, and *H. lixii* F3ST1) have demonstrated high colonization rate of the maize host plant parts through seed inoculation and were therefore selected for their potential insecticidal activity against the developmental stages (oviposition, larval and pupal mortality/survival, and adult emergence) of FAW.

To assess the effect of endophytic colonization of maize on the oviposition potential of *S. frugiperda*, seeds were inoculated with the respective isolates and sowed in sterile planting substrate (a mixture of manure and soil at a ratio of 1:5) as described above. Two-week-old endophytically-colonized maize seedlings were exposed to four-day-old, mated moths (10 individuals at sex ratio of 1:2 male:female) for 48 h in Plexiglas cages (50 × 50 × 60 cm). Each treatment consisted of a total of six potted maize plants which was replicated four times. All the treatments were maintained at 25°C ± 2°C and were arranged in a randomized complete block design. After 48 h, moths were removed from the exposure cages and introduced into clean cages (50 × 50 × 60 cm). Moths were fed with 10% sugar solution soaked in balls of cotton wool as food source (Akutse et al., 2019). The number of egg masses per plant was counted and leaves with egg mases were cut off from these plants and then incubated in a clean sterile plastic container (21 cm long × 15 cm wide × 8 cm high) until the eggs hatched.

After hatching, 50 larvae (neonates) were randomly picked using a fine camel hairbrush and then transferred into individual Petri dishes lined with filter paper. For each treatment, ten FAW neonates were placed on the adaxial side of endophytically-colonized maize leaf disks (3 × 3 cm) in a Petri dish and sealed with Parafilm membrane. In the control, FAW neonates were fed on endophyte-free maize leaf disks (Russo et al., 2021). This experiment was replicated four times. Larvae were allowed to feed on the maize leaf disks and leaf defoliation was recorded until they pupated. For each treatment, pupation was recorded daily, and pupae were then incubated at 25°C ± 2°C. Subsequently, the emergence of the moths was determined for each treatment, and non-viable pupae were also counted and dissected under microscope to assess the actual viability. Following adult emergence from the endophytically-colonized and control plants (F1 progeny), moths were collected per treatment and their sex ratio was determined/recorded and this was replicated four times (Akutse et al., 2013). The F1 moths were maintained in a cage as described above and were fed with 10% sugar solution soaked in balls of cotton wool as food source and the cage was maintained at 25°C ± 2°C, 48%–60% RH and 12:12L:D photoperiod. Mycosis test was performed to confirm if the mortality of the larvae, pupa and adult moths was due to direct fungal infection. Therefore, dead insects were placed on a sterile moistened filter paper in Petri dishes and were observed for post-mortem fungal sporulation. All the dead insects were surface sterilized with 1% sodium hypochlorite and rinsed three times with sterile distilled water prior to incubation. The surface sterilized cadavers were placed on wet filter paper in sterile Petri dishes that were then sealed with Parafilm membrane and kept at room temperature (Akutse et al., 2013; Agbessenou et al., 2020). The experiments were replicated four times.

### 2.7 Statistical analyses

All analyses were performed in R software version 3.6.2 (R Core Team, 2019). Normality and homogeneity of variance tests were performed on colonization rate and count data (number of egg masses, number of pupae and number of adults) using Shapiro-Wilk test (Shapiro and Wilk, 1965) and Levene test, respectively. Since colonization data were not normally distributed, and the variances were not homogeneous; therefore, the data were analyzed with generalized linear model (GLM) using binomial distribution and logit link function (Agbessenou et al., 2020). Count data were analyzed with GLM assuming a negative binomial error distribution and the means were separated using Tukey’s honest significant difference (HSD) test (*p* < 0.05) (Muvea et al., 2014). Percentage leaf defoliation was analyzed with beta regression, while the plant height, leaf length, leaf width, wet weight and dry shoot were analyzed using analysis of variance (ANOVA), and the differences in means were separated using Student-Newman-Keuls (SNK) test (Paradza et al., 2021).

## 3 Results

### 3.1 Endophytic colonization of maize plants by fungal isolates

**FIGURE 1:**
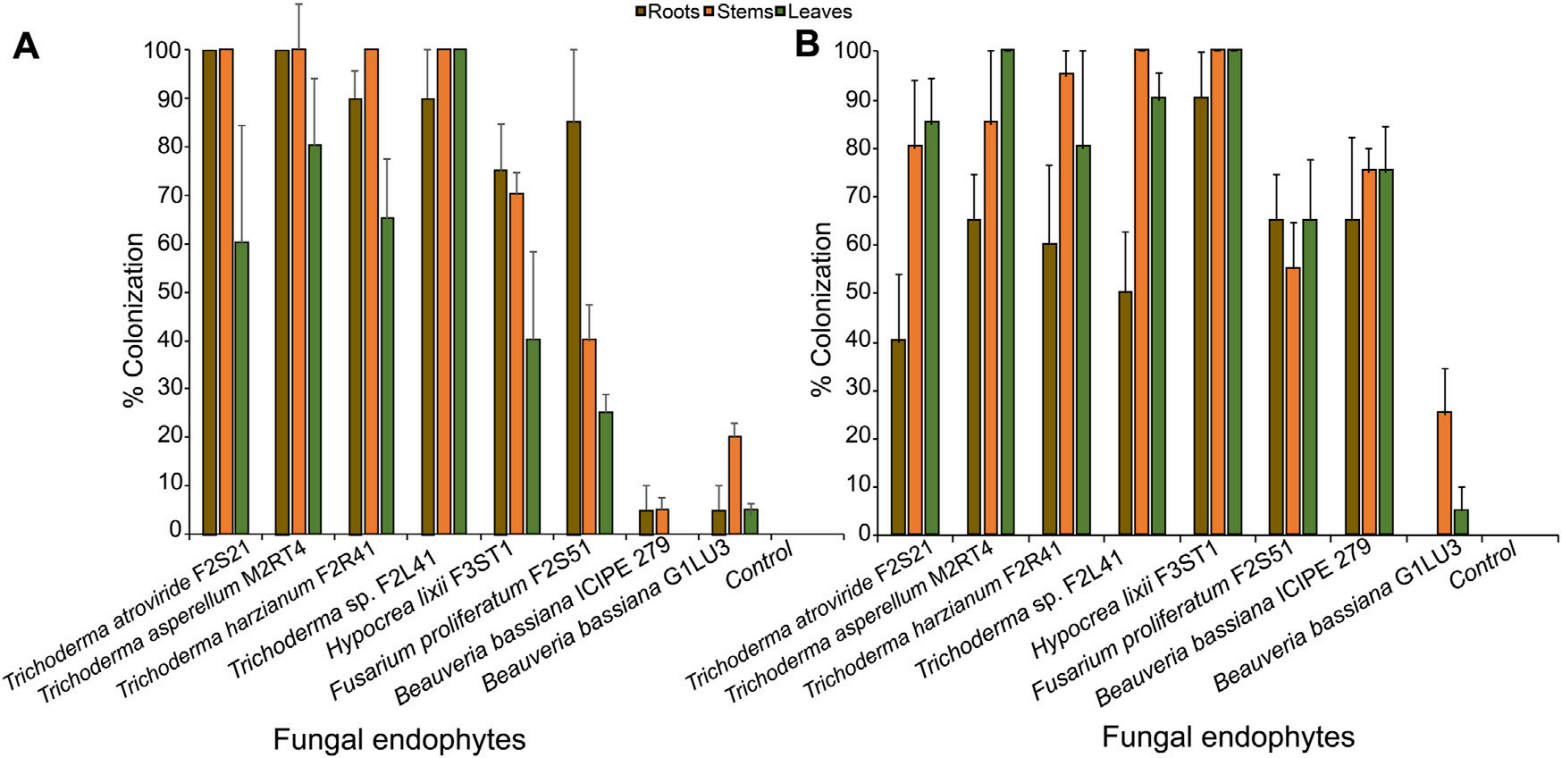
Endophytic colonization of different parts of maize host plants through seed inoculation **(A)** and foliar inoculation **(B)** by eight fungal isolates. Bar chart represents means ± SE (standard error) at 95% CI (*p* < 0.05; *n* = 4).

In this study, the results on viability test showed that conidia germination of the different fungal isolates exceeded 90% after 18 h of incubation. Fourteen days post-inoculation, all the tested isolates were successfully recovered as endophytes from inoculated plants (Figures 1A, B). However, the extent of colonization of different parts of the maize plant (roots, stems, and leaves) varied depending on the inoculation method. There was significant effect of the inoculation method on the colonization of the leaves (W = 581, *p* = 0.40) but not the roots (W = 354.5; df = 1; *p* = 0.15) and the stems (W = 566.5; df = 1; *p* = 0.44). Additionally, the colonization rate highly depended on the fungal isolates and plant parts. For example, *Trichoderma* sp. F2L41 had the highest percentage colonization of the leaves (100%); whereas *T*. *atroviride* F2S21 and *T. asperellum* M2RT4 had the highest colonization of the roots (100%) through seed inoculation. Further, *T. atroviride* F2S21, *T. asperellum* M2RT4, *T. harzianum* F2R41, and *Trichoderma* sp. F2L41 isolates had the highest rate of stem colonization after seed inoculation (Figure 1A). Through seed inoculation, *B. bassiana* ICIPE 279 was not able to colonize the leaf tissues of the host plant, whereas foliar application resulted in colonization of all plant tissues by the same isolate. *Hypocrea lixii* F3ST1 exhibited the highest colonization rate with 90, 100% and 100% of roots, stems and leaves, respectively, through leaf inoculation. Additionally, *B*. *bassiana* G1LU3 only colonized stem (25%) and leaf (5%) tissues through foliar application, while it colonized roots (5%), stems (20%) and leaves (5%) through seed inoculation (Figures 1A, B).

### 3.2 Endophytic colonization persistence for selected isolates

**FIGURE 2:**
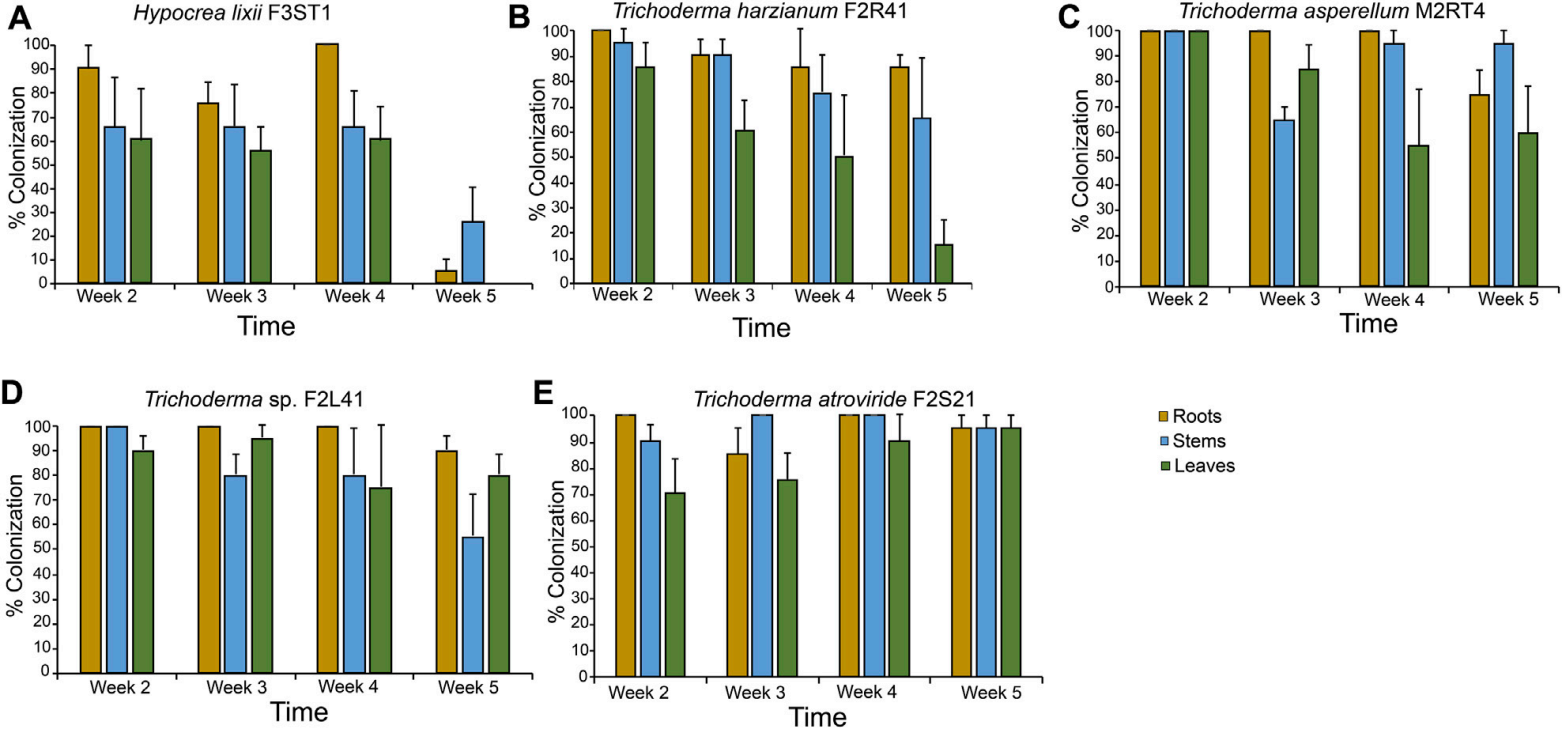
Endophytic colonization persistence of *Hypocrea lixii* F3ST1 **(A)**, *Trichoderma harzianum* F2R41 **(B)**, *Trichoderma asperellum* M2RT4 **(C)**, *Trichoderma* sp. F2L41 **(D)**, and *Trichoderma atroviride* F2S21 **(E)** 5 weeks post-germination. Bar chart represents means ± SE (standard error) at 95% CI (*p* < 0.05; *n* = 4).

Based on their ability to highly colonize all the plant parts, *T. atroviride* F2S21, *T. harzianum* F2R41, *Trichoderma* sp. F2L41, *T. asperellum* M2RT4, and *H. lixii* F3ST1 were selected to assess the persistence of their colonization of maize host plant through seed inoculation. There were significant interactions between the isolates and the time in the roots (*χ* ^2^ = 51.59, df = 12, *p* < 0.001), stems (*χ* ^2^ = 22.93, df = 12, *p* = 0.02) and leaves (*χ* ^2^ = 27.04, df = 12, *p* < 0.01) (Figure 2). During the first week, percentage colonization of the plant parts ranges between 90%–100%, 65%–100%, and 60%–100%, in the roots, stems, and leaves, respectively for all the five isolates. The colonization pattern significantly decreased over time for *H. lixii* F3ST1 (stems: *χ* ^2^ = 1.98, df = 3, *p* = 0.04 and leaves: *χ* ^2^ = 17.97, df = 3, *p* < 0.001), for *T. harzianum* F2R41 (leaves: *χ* ^2^ = 10.44, df = 3, *p* = 0.01) and for *T. asperellum* M2RT4 (roots: *χ* ^2^ = 61.01, df = 3, *p* < 0.001) (Figure 2). In the final week (week five), there was no presence of *H. lixii* F3ST1 in the leaves while it was 5% and 25% in the roots and stems, respectively (Figure 2).

### 3.3 Effect of endophytic colonization on maize seedling growth parameters

**FIGURE 3:**
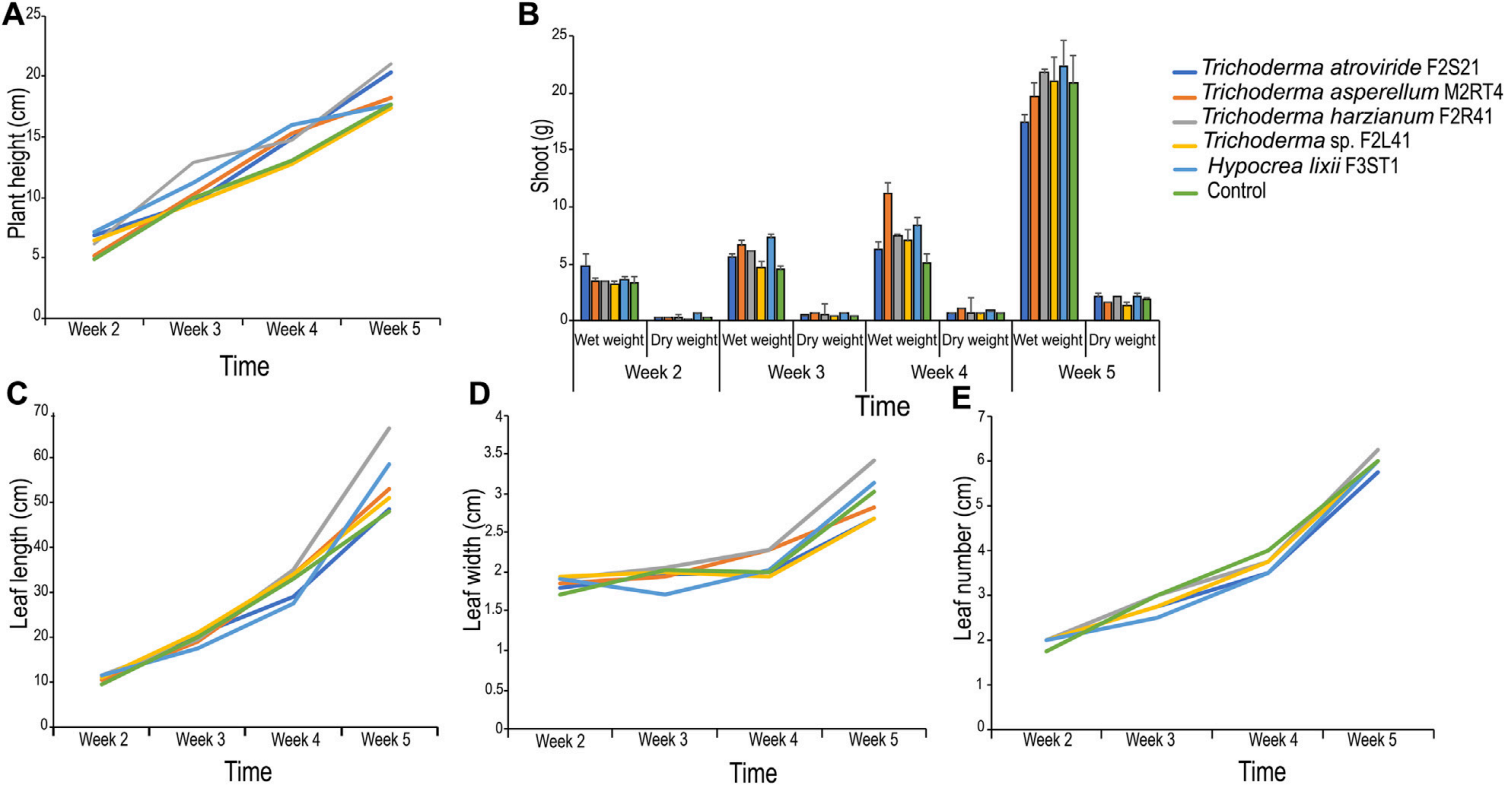
Effect of endophytic colonization of the five fungal isolates on maize seedling growth parameters. **(A)** Mean plant height; **(B)** Mean wet and dry shoot weight; **(C)** Mean leaf length; **(D)** Mean leaf width and **(E)** Mean leaf number.

The weekly assessment showed varied effects of the endophytes on maize plant growth parameters compared to the control (Figure 3). For example, there was no significant difference in plant height in the second and the third week post-germination. However, significant difference in plant height (F = 6.88, df = 5, *p* < 0.001) was observed in the fourth week among the treatments with *T. harzianum* F2R41 outperforming all the other isolates (Figure 3A).

In addition, there was no significant difference in wet shoot weight (F = 1.01, df = 5, *p* = 0.439) among the treatments in the second week; while significant difference in wet shoot weight (F = 6.29, df = 5, *p* < 0.01) was observed in the third week with *H. lixii* F3ST1 and *T. asperellum* M2RT4 outperforming all the other isolates (Figure 3B). In the fourth week, significant difference in wet shoot weight (F = 5.73, df = 5, *p* < 0.01) was observed among the treatments with *T. asperellum* M2RT4 outperforming all the other isolates. There was also significant difference in dry shoot weight (F = 3.06, df = 5, *p* = 0.03) among the treatments in the second week with *H. lixii* F3ST1 recording the highest value (0.59 ± 0.19 g) (Figure 3B). In the third week, significant difference in dry shoot weight (F = 11.04, df = 5, *p* < 0.001) was observed among the treatments with *H. lixii* F3ST1 and *T. asperellum* M2RT4 outperforming all the other isolates (Figure 3B); while in the fourth week *T asperellum* M2RT4 exhibited the highest dry shoot weight (F = 7.51, df = 5, *p* < 0.001) compared to the other treatments (Figure 3B).

No significant difference in leaf length was observed among the treatments in the second- and third-week post-inoculation (Figure 3C). However, in the fifth week, the leaf length was significantly (F = 4.17, df = 5, *p* < 0.01) higher in *T. harzianum* F2R41 than in the other treatments (Figure 3C). Similarly, no significant differences in leaf width were observed among the treatments in the second- and third-week post-inoculation (Figure 3D). However, in the fourth week, the leaf width was significantly (F = 3.88, df = 5, *p* < 0.01) higher in *T. harzianum* F2R41 and in *T. asperellum* M2RT4 than in the other treatments (Figure 3D). On the other hand, there was no significant difference in the number of leaves among the treatments in all the weeks of assessment (Figure 3E).

### 3.4 Effect of endophytic colonization of maize plants on egg masses of *Spodoptera frugiperda*

**FIGURE 4:**
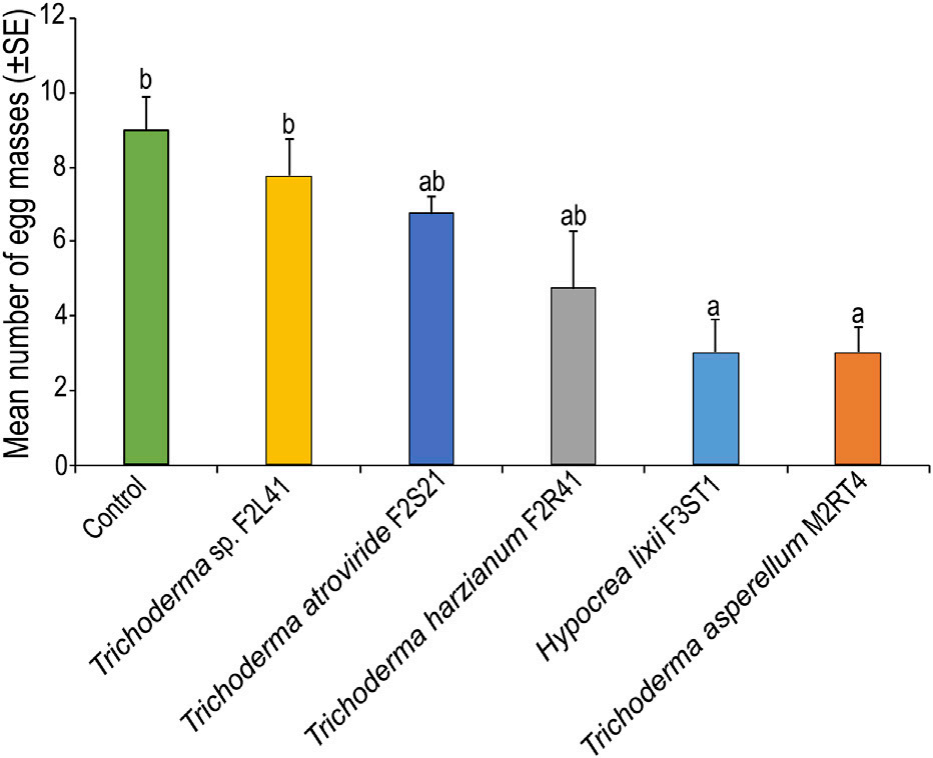
Effect of endophytically-colonized maize plants on egg masses of *Spodoptera frugiperda*. Bar chart represents means ± SE (standard error) at 95% CI (*p* < 0.05; *n* = 4).

The number of egg masses laid on endophytically-colonized maize plants varied among the treatments (*χ* ^2^ = 22.93, df = 5, *p* < 0.001) (Figure 4). For instance, *T. asperellum* M2RT4 endophytically-colonized maize plants recorded the lowest number of egg masses (3 ± 0.70 egg masses), followed by *H. lixii* F3ST1 (3 ± 0.91 egg masses) compared to 9 ± 0.91 egg masses in the control (Figure 4).

### 3.5 Effect of endophytic colonization of maize plants on leaf defoliation of *Spodoptera frugiperda*

**FIGURE 5:**
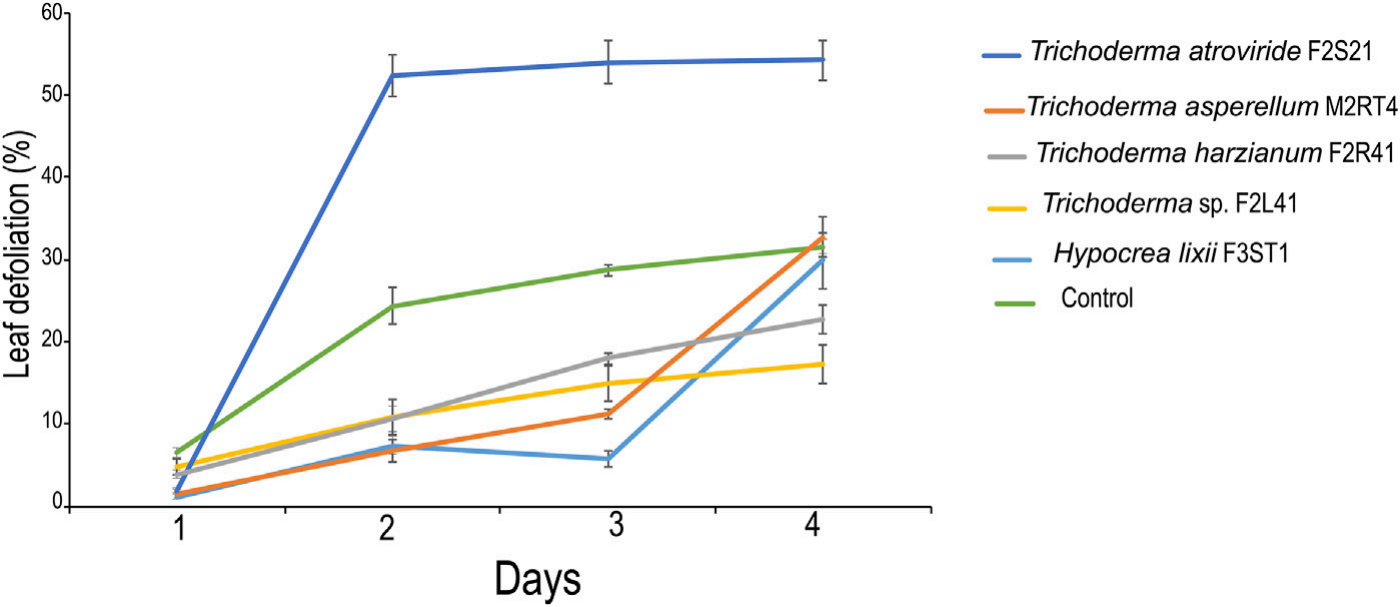
Endophytic colonization of maize plants on leaf defoliation or feeding rate of *Spodoptera frugiperda*.

At day 1, leaf defoliation rate was significantly lower in *H. lixii* F3ST1, followed by *T. asperellum* M2RT4 and *T. atroviride* F2S21 than in the control (χ^2^ = 71.66, df = 5, *p* < 0.001) (Figure 5). At day 2, the leaf defoliation was significantly lower in *T. asperellum* M2RT4 and *H. lixii* F3ST1 compared to the control (*χ* ^2^ = 17.07, df = 5, *p* < 0.01); while the highest percentage leaf defoliation was recorded on *T. atroviride* F2S21 (Figure 5). At day 3, there was a significant difference in leaf defoliation among the treatments (*χ* ^2^ = 17.08, df = 5, *p* < 0.01) with the lowest percentage defoliation recorded on *H. lixii* F3ST1, while the highest was obtained on *T. atroviride* F2S21 (Figure 5). However, at day 4, there was no significant difference in leaf defoliation among the treatments (*χ* ^2^ = 2.80, df = 5, *p* = 0.73) (Figure 5).

### 3.6 Effect of endophytic colonization of maize plants on pupation and adult emergence of *Spodoptera frugiperda*

**FIGURE 6:**
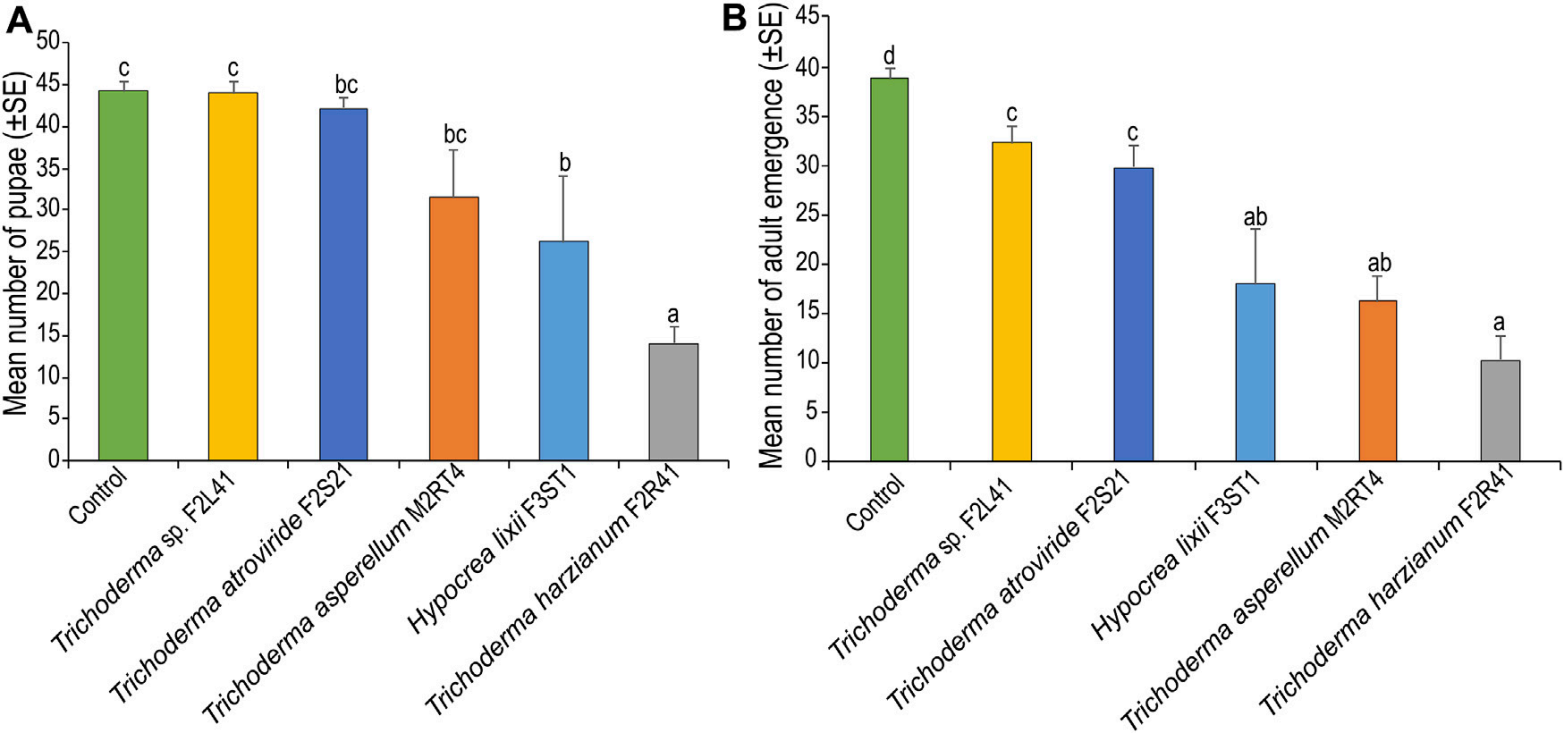
Endophytic colonization of maize plants on **(A)** pupation and **(B)** adult emergence of Spodoptera frugiperda. Bar chart represents means ± SE (standard error) at 95% CI (*p* < 0.05; *n* = 4).

There was a significant difference in pupal formation among the treatments (*χ* ^2^ = 53.58, df = 5, *p* < 0.001), where fewer pupae (14 ± 2.12 pupae) were recorded in *T. harzianum* F2R41, followed by *H. lixii* F3ST1 (26.25 ± 7.79 pupae) compared to the control (44.25 ± 1.10 pupae) (Figure 6A). Further, *S. frugiperda* adult emergence varied significantly among the treatments (*χ* ^2^ = 33.89, df = 5, *p* < 0.001), with the lowest number of adults (10.25 ± 2.52) recorded in *T. harzianum* F2R41, followed by *T. asperellum* M2RT4 (16.25 ± 2.49 moths) compared to the control (38.75 ± 1.10 moths) (Figure 6B).

### 3.7 Sex ratio

A significantly female-biased sex ratio was observed (*χ* ^2^ = 35.24, df = 5, *p* < 0.001) in the emerging moths among the treatments. For instance, the sex ratio was (1:1.58), (1:1.73), (1:1.30), and (1:1.88) (males: females) in the control, *T. harzianum* F2R41, *Trichoderma* sp. F2L41, and *H. lixii* F3ST1, respectively, unlike in *T. atroviride* F2S21 and *T. asperellum* M2RT4 where the sex ratio was balanced 1:1 (Table 1).

**TABLE 1:** Sex ratio (males:females) of *Spodoptera frugiperda* individuals that emerged from endophytically-colonized maize plants.

Fungal species	Isolates	Sex ratio (males:females)
*Trichoderma harzianum*	F2R41	1:1.73
*Trichoderma* sp	F2L41	1:1.30
*Hypocrea lixii*	F3ST1	1:1.88
*Trichoderma atroviride*	F2S21	1.13:1
*Trichoderma asperellum*	M2RT4	1:1
Control	-	1:1.58

## 4 Discussion

Our study demonstrates the potential of *T. atroviride* F2S21, *T. asperellum* M2RT4, *T. harzianum* F2R41, *Trichoderma* sp. F2L41, *H. lixii* F3ST1 and *F. proliferatum* F2S51 to systemically colonize different maize host plant parts and improve plant growth through seed treatment. The results showed that *T. asperellum* M2RT4, *T. harzianum* F2R41, *Trichoderma* sp. F2L41, and *H. lixii* F3ST1 negatively affect the life-history parameters of *S. frugiperda* which is of crucial importance in the design of control strategies for the sustainable management of the pest.

We found that the extent of colonization of different parts of maize host plant (roots, stems, and leaves) varied depending on the inoculation method and fungal isolates. Also, our findings revealed that *T. atroviride* F2S21, *T. asperellum* M2RT4, *T. harzianum* F2R41, *Trichoderma* sp. F2L41, *H. lixii* F3ST1 and *F. proliferatum* F2S51 colonized roots, stems, and leaves of the plants through both seed and foliar inoculation methods. Similar results were obtained for *T. atroviride* F2S21, *T. asperellum* M2RT4, *T. harzianum* F2R41, *Trichoderma* sp. F2L41, *H. lixii* F3ST1 whose endophytic ability was demonstrated in tomato and nightshade (Agbessenou et al., 2020) and in French bean (*P. vulgaris* L.) (Paradza et al., 2021). The successful colonization of maize host plants by the endophytes suggests that these fungal species have escaped the immune signal of the host plant and therefore penetrating tissues to become symbiotic within maize host plant. In addition, *H*. *lixii* F3ST1 exhibited the highest colonization rate of maize plant parts through foliar inoculation. This supports the observation that not all endophytic fungal species show the same endophytic capabilities. In a previous study, Mutune et al. (2016) also reported on the higher percentage colonization of roots and stems of the common bean, *Phaseolus vulgaris* by *H. lixii* F3ST1. Through seed inoculation, *B. bassiana* ICIPE 279 was not able to colonize the leaf tissues of the host plant, whereas the isolate was recovered from all the plant parts through foliar application. This supports the hypothesis that the success of colonization depends on the compatibility between the host plant and the endophyte species (Jaber, 2015; Paradza et al., 2021). Conversely, Russo et al. (2021) demonstrated that the highest colonization rate of maize host plant parts was obtained through foliar inoculation of *B. bassiana* isolate LPSc 1098. Besides, Jaber and Ownley (2018) speculated that the differential colonization rates could be attributed to the chemistry on the leaf surface or competition with other endophytes naturally occurring within host plants.

Our results indicated that successful colonization of maize host plant by the various endophytic fungal species depends on the plant part, the persistence time which significantly influenced the outcome of the colonization. Interestingly, we established that successful colonization of maize plant parts by the best performing isolates (*T. atroviride* F2S21, *T. asperellum* M2RT4, *T. harzianum* F2R41, *Trichoderma* sp. F2L41, *H. lixii* F3ST1 and *F. proliferatum* F2S51) was achieved during the first week post-inoculation. This confirms the observation that upon colonization, the fungal inoculum requires time to move through intercellular tissues so as to colonize the whole plant (Paradza et al., 2021). Subsequently, the colonization pattern significantly decreased over time for *H. lixii* F3ST1 in both the stems and the leaves, for *T. harzianum* F2R41 in the leaves and for *T. asperellum* M2RT4 in the roots. In the final week (week five), there was no presence of *H. lixii* F3ST1 in the leaves while it was 5% and 25% in the roots and stems, respectively. Our findings are in agreement with other studies that reported that the extent and persistence of plant colonization with endophytic fungal isolates is influenced by the fungal strain, host plant species and parts (Bamisile et al., 2020; Paradza et al., 2021).

The weekly measurements of various growth parameters did not show significant growth promotions in the colonized plants in some traits compared to the control. However, the effect of *H. lixii* F3ST1 in enhancing growth in maize plant was evident in the shoot biomass accumulated during the entire growing period. *Hypocrea lixii* F3ST1 induced the highest fresh and dry shoot weight compared to other treatments. Previously, Jaber and Enkerli (2016) demonstrated the ability of fungal entomopathogens to promote plant growth following successful endophytic establishment within broad bean *Vicia faba*. The same authors further speculated that the enhanced plant growth observed in maize plant might be attributed to the production of phytohormones. Also, Paradza et al. (2021) reported that the endophytic fungal isolate *T*. *atroviride* F5S21 gave the highest fresh and dry shoot weight compared with other treatments in tomato. Besides, plant growth promotion induced by these endophytic fungal isolates could be due to the increased uptake of nutrients (Behie et al., 2017).

In this study, the insecticidal activity of selected endophytic isolates was assessed on the life-history parameters of *S. frugiperda*. We observed a significant reduction in the number of egg masses laid on *T. asperellum* M2RT4 endophytically-colonized maize plants, indicating that the female of *S. frugiperda* might prefer host plants on which their offspring will perform/develop better. The search for a suitable host plant for oviposition is crucial for the fitness of herbivorous insects as it affects the survival of their offspring (Nataraj et al., 2021). Agbessenou et al. (2020) previously reported significant reduction in oviposition on tomato and nightshade host plants mediated by the endophyte *T. asperellum* M2RT4. Similarly, Paradza et al. (2021) demonstrated that *T. asperellum* M2RT4 endophytically-colonized French bean plants significantly reduce oviposition of the greenhouse whiteflies, *Trialeurodes vaporariorum* Westwood (Hemiptera: Aleyrodidae). In addition, the findings showed significant effect of *H. lixii* F3ST1, followed by *T. asperellum* M2RT4 endophytically-colonized maize plants on the defoliation rates of immature stages of the pest which could be attributed to antifeedant or deterrent effects mediated by the production of secondary metabolites in the colonized host plants (Russo et al., 2021). Conversely, we recorded the highest percentage defoliation on *T. atroviride* F2S21 endophytically-colonized maize plants. This is quite surprising as the presence of the fungal inoculum in the leaves would have prevented the larvae from feeding on the colonized plants; we therefore hypothesized that the nutritional composition of the colonized maize plants by *T. atroviride* F2S21 might make them highly suitable for the larvae to continue voraciously feeding on the host, calling for further studies to elucidate this feeding behaviour of the pest.

Furthermore, our findings also indicated that *T. harzianum* F2R41 had the highest overall negative impact on the number of pupae and adult emergence of *S. frugiperda*. Similar results were reported by Paradza et al. (2021) who recorded negative effect of *T. harzianum* F2R41 on the oviposition and the development of nymphs to the fourth instar of the greenhouse whitefly, *T*. *vaporariorum* feeding on tomato and French bean endophytically-colonized by F2R41. Even though there was strong evidence of mortality of insects feeding on endophytically-colonized plants, there was no signs of fungal outgrowth on the cadavers indicating that the cause of death of the insects remains elusive. However, it has been hypothesized that negative effects on herbivorous insects could be as a result of antibiosis and feeding deterrence mediated by the presence of the endophytes *in planta*, triggering the production of secondary metabolites (Akello and Sikora, 2012). Thus, both host plants and endophytic fungal isolates exhibited symbiotic relationship which allows both partners to evolve strategies such as chemical and molecular mechanisms for mutual adaptation following *S. frugiperda* herbivory. The results obtained corroborate the hypothesis that resistance could be induced and enhanced against *S. frugiperda* upon endophytic plant colonization (Russo et al., 2021). The enhanced protection in maize plant could therefore most likely due to the activation of different signalling pathways triggered by the presence of endophytic fungal isolates. In this study, we further observed a female-biased sex ratio which concurs with findings of Sokame et al. (2020) who also reported female-biased sex ratio in fall armyworm population, therefore supporting the hypothesis that females fall armyworm tend to sustain the progeny of the pest under unfavourable conditions. Conversely, Akutse et al. (2013) found a male-biased sex ratio in *Liriomyza huidobrensis* populations (Diptera: Agromyzidae) where more males emerged from fungus-infected host plants than uninfected plants.

In this study, we have identified *T. asperellum* M2RT4, *T. harzianum* F2R41, and *H. lixii* F3ST1 as the best performing endophytic fungal isolates improving maize plant defense against *S. frugiperda* and this was evidenced with the reduction of adult oviposition, leaf defoliation, pupation and adult emergence as compared to other treatments. Therefore, the potential use of these isolates as endophytic-based biopesticides could be considered as part of a sustainable *S. frugiperda* management strategy in maize production systems. Additionally, *T. asperellum* M2RT4, *T. harzianum* F2R41, and *H. lixii* F3ST1 could be integrated with other biocontrol agents to promote a comprehensive IPM strategy against FAW. However, further studies need to be conducted to elucidate the underlying mechanisms by which the presence of these potent endophytes within maize host plants affect *S. frugiperda* along with the validation of the findings under field conditions.

## Data Availability

The original contributions presented in the study are included in the article/Supplementary material, further inquiries can be directed to the corresponding author.
